# 4-Amino-3,5-di-2-pyridyl-4*H*-1,2,4-triazole

**DOI:** 10.1107/S1600536808025658

**Published:** 2008-08-16

**Authors:** Manuela Ramos Silva, Joana A. Silva, Nuno D. Martins, Ana Matos Beja, Abilio J. F. N. Sobral

**Affiliations:** a CEMDRX, Physics Department, University of Coimbra, P-3004-516 Coimbra, Portugal; b Chemistry Department, University of Coimbra, P-3004-516 Coimbra, Portugal

## Abstract

In the crystal structure of the title compound, C_12_H_10_N_6_, the mol­ecules deviate slightly from planarity. The plane of the central triazole ring makes angles of 6.13 (9) and 3.28 (10)° with the pyridyl ring planes. Intra­molecular N—H⋯N inter­actions form six-membered closed rings. The crystal packing also shows weak C—H⋯π and C—H⋯N inter­actions.

## Related literature

For related literature, see: Dirtu *et al.* (2007 ▶); Faulmann *et al.* (1990 ▶); Haasnoot (2000 ▶); van Koningsbruggen *et al.* (1995 ▶); Malone *et al.* (1997 ▶), Mernari *et al.* (1998 ▶).
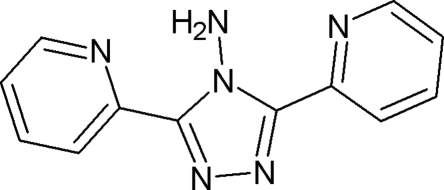


## Experimental

### 

#### Crystal data

C_12_H_10_N_6_
*M* *_r_* = 238.26
Monoclinic, 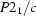

*a* = 6.6191 (2) Å
*b* = 14.7136 (4) Å
*c* = 11.4703 (4) Å
β = 95.474 (2)°
*V* = 1112.01 (6) Å^3^
*Z* = 4
Mo *K*α radiation
μ = 0.09 mm^−1^
*T* = 293 (2) K
0.20 × 0.13 × 0.12 mm

#### Data collection

Bruker APEX CCD area-detector diffractometer
Absorption correction: multi-scan (*SADABS*; Sheldrick, 2000 ▶) *T*_min_ = 0.91, *T*_max_ = 0.99
23253 measured reflections
2751 independent reflections
1465 reflections with *I* > 2σ(*I*)
*R*_int_ = 0.052

#### Refinement

*R*[*F* ^2^ > 2σ(*F* ^2^)] = 0.045
*wR*(*F* ^2^) = 0.116
*S* = 1.00
2751 reflections
193 parameters
Only H-atom coordinates refined
Δρ_max_ = 0.19 e Å^−3^
Δρ_min_ = −0.15 e Å^−3^

### 

Data collection: *SMART* (Bruker, 2003 ▶); cell refinement: *SAINT* (Bruker, 2003 ▶); data reduction: *SAINT*; program(s) used to solve structure: *SHELXS97* (Sheldrick, 2008 ▶); program(s) used to refine structure: *SHELXL97* (Sheldrick, 2008 ▶); molecular graphics: *ORTEPII* (Johnson, 1976 ▶); software used to prepare material for publication: *SHELXL97*.

## Supplementary Material

Crystal structure: contains datablocks global, I. DOI: 10.1107/S1600536808025658/bx2169sup1.cif

Structure factors: contains datablocks I. DOI: 10.1107/S1600536808025658/bx2169Isup2.hkl

Additional supplementary materials:  crystallographic information; 3D view; checkCIF report

## Figures and Tables

**Table 1:** Hydrogen-bond geometry (Å, °) *Cg*1 and *Cg*2 are the centroids of the N6/C8–C12 and N5/C3–C7 rings, respectively.

*D*—H⋯*A*	*D*—H	H⋯*A*	*D*⋯*A*	*D*—H⋯*A*
N4—H4*A*⋯N6	0.91 (2)	2.08 (2)	2.839 (2)	141.1 (17)
N4—H4*B*⋯N5	0.87 (2)	2.39 (2)	2.863 (2)	115.0 (16)
C4—H4⋯N5^i^	0.990 (18)	2.509 (19)	3.457 (2)	160.1 (14)
C7—H7⋯N3^ii^	0.988 (19)	2.58 (2)	3.444 (2)	146.2 (14)
C6—H6⋯*Cg*1^iii^	0.92 (2)	2.75 (2)	3.504 (2)	140.4 (16)
C11—H11⋯*Cg*2^iv^	1.00 (2)	2.86 (2)	3.602 (2)	131.9 (17)

Symmetry codes: (i) 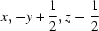; (ii) 
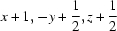
; (iii) 
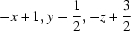
; (iv) 
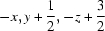
.

**Fig. 1.**
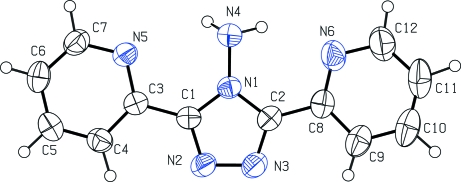
ORTEPII (Johnson, 1976) plot of the title compound. Displacement ellipsoids are drawn at the 50% level.

**Fig. 2.**
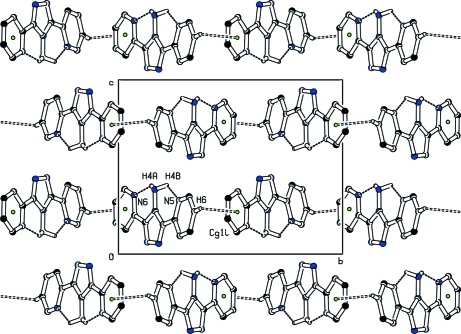
Packing diagram of the title compound, showing the N—H···N intramolecular hydrogen bonds and the C—H···π intermolecular interactions as dashed lines. The green dot represents the centroid of the N6—C8—C9—C10—C11—C12 ring.

## supplementary crystallographic information

### Comment

1,2,4-triazoles and its derivatives have proven to be good multi-N-donor ligands (Haasnoot, 2000) coordinating first row transition metals in mononuclear complexes or bridging the metal atoms into polymeric complexes. Some of those complexes display interesting magnetic properties, for example, chains of Fe(II)-4-amino-1,2,4-triazole exhibit hysteretic spin transitions at around 200 K and dinuclear copper-4-amino-3,5bis(pyridin-2-yl) -1,2,4-triazole clusters order antiferromagnetically above 50 K (Dirtu *et al.*, 2007; Koningsbruggen *et al.*, 1995). Within a project of synthesizing new metal-triazole complexes, we have obtained crystals of the title compound. The 4-Amino-3,5-bis(pyridin-2-yl)-1,2,4-triazole molecules, I, Fig. 1, deviate slightly from planarity, as seen by the torsion angles N3—C2—C8—C9 - 2.7 (3) and C4—C3—C1—N2 4.6 (2)°. The molecule has a pseudo-twofold axis bisecting the triazole ring. Both H atoms of the amino group participate in intramolecular H-bonds with the pyridyl N atoms as acceptors, defining the molecule conformation (Table 1). In metal complexes, the pyridyl rings often rotate around the single C—C bond so that the pyridyl N atom can coordinate the metal ion, for instance like in 4-amino-3,5-bis(pyridin-2-yl)-1,2,4-triazole diaqua manganese dibromide (Faulmann *et al.* 1990).

C—H···π intermolecular interactions also occur between these organic molecules. One pyridyl carbon at each side of the molecule directs its H atom towards the electron cloud of neighbouring pyridyl aromatic rings linking the molecules together (Fig. 2). The geometry of the interaction corresponds to the type III as classified by Malone *et al.* (1997), with the H atoms positioned almost above the centroid of the acceptor ring and the C—H bond pointing towards the edge of the ring [C6···*Cg*1^i^ 3.504 (2) ° and C6—H6···*Cg*1^i^ angle 140.4 (16)°, i:1 - *x*,-1/2 + *y*,3/2 - *z* and *Cg*1 is the centroid of the six-membered ring N6—C8—C9—C10—C11—C12]. There is a similar interaction on the other end of the molecule (C11) but with a longer distance [3.602 (2) Å].

### Experimental

0.02 mmol (0.005 g) of 4-amino-3,5-bis(pyridin-2-yl)-1,2,4-triazole were dissolved in 5 ml of dimethylformamide. 0.006 mmol (0.003 g) of NH_4_Fe(SO_4_)_2_.12H_2_O were then added to the solution.

### Refinement

The H atoms were located in Fourier difference maps and their coordinates were freely refined. Isotropic displacement factors were used for all H atoms with *U*_iso_(H)=1.2*U*_eq_(parent atom).

### Crystal data

**Table:**

C_12_H_10_N_6_	*F*_000_ = 496
*M**_r_* = 238.26	*D*_x_ = 1.423 Mg m^−^^3^
Monoclinic, *P*2_1_/*c*	Mo *K*α radiation λ = 0.71073 Å
Hall symbol: -P 2ybc	Cell parameters from 3853 reflections
*a* = 6.6191 (2) Å	θ = 2.3–22.9º
*b* = 14.7136 (4) Å	µ = 0.09 mm^−^^1^
*c* = 11.4703 (4) Å	*T* = 293 (2) K
β = 95.474 (2)º	Block, colourless
*V* = 1112.01 (6) Å^3^	0.20 × 0.13 × 0.12 mm
*Z* = 4	

### Data collection

**Table:**

Bruker APEX CCD area-detector diffractometer	2751 independent reflections
Radiation source: fine-focus sealed tube	1465 reflections with *I* > 2σ(*I*)
Monochromator: graphite	*R*_int_ = 0.052
*T* = 293(2) K	θ_max_ = 28.4º
φ and ω scans	θ_min_ = 2.3º
Absorption correction: multi-scan(SADABS; Sheldrick, 2000)	*h* = −8→8
*T*_min_ = 0.91, *T*_max_ = 0.99	*k* = −19→19
23253 measured reflections	*l* = −15→14

### Refinement

**Table:**

Refinement on *F*^2^	Secondary atom site location: difference Fourier map
Least-squares matrix: full	Hydrogen site location: inferred from neighbouring sites
*R*[*F*^2^ > 2σ(*F*^2^)] = 0.045	Only H-atom coordinates refined
*wR*(*F*^2^) = 0.116	*w* = 1/[σ^2^(*F*_o_^2^) + (0.046*P*)^2^ + 0.1952*P*] where *P* = (*F*_o_^2^ + 2*F*_c_^2^)/3
*S* = 1.00	(Δ/σ)_max_ < 0.001
2751 reflections	Δρ_max_ = 0.19 e Å^−^^3^
193 parameters	Δρ_min_ = −0.15 e Å^−^^3^
Primary atom site location: structure-invariant direct methods	Extinction correction: none

### Special details

**Table:**

Geometry. All e.s.d.'s (except the e.s.d. in the dihedral angle between two l.s. planes) are estimated using the full covariance matrix. The cell e.s.d.'s are taken into account individually in the estimation of e.s.d.'s in distances, angles and torsion angles; correlations between e.s.d.'s in cell parameters are only used when they are defined by crystal symmetry. An approximate (isotropic) treatment of cell e.s.d.'s is used for estimating e.s.d.'s involving l.s. planes.
Refinement. Refinement of *F*^2^ against ALL reflections. The weighted *R*-factor *wR* and goodness of fit *S* are based on *F*^2^, conventional *R*-factors *R* are based on *F*, with *F* set to zero for negative *F*^2^. The threshold expression of *F*^2^ > σ(*F*^2^) is used only for calculating *R*-factors(gt) *etc*. and is not relevant to the choice of reflections for refinement. *R*-factors based on *F*^2^ are statistically about twice as large as those based on *F*, and *R*- factors based on ALL data will be even larger.

### Fractional atomic coordinates and isotropic or equivalent isotropic displacement parameters (Å^2^)

**Table:**

	*x*	*y*	*z*	*U*_iso_*/*U*_eq_	
C2	0.1009 (3)	0.37992 (12)	0.68150 (16)	0.0427 (4)	
C7	0.7970 (3)	0.18467 (13)	0.82952 (18)	0.0530 (5)	
H7	0.836 (3)	0.1715 (13)	0.9131 (17)	0.064*	
N1	0.2528 (2)	0.33629 (9)	0.74750 (12)	0.0384 (4)	
C3	0.5629 (2)	0.24896 (11)	0.69618 (15)	0.0379 (4)	
N5	0.6192 (2)	0.22823 (10)	0.80804 (12)	0.0465 (4)	
N2	0.2987 (2)	0.32021 (11)	0.56204 (13)	0.0501 (4)	
N4	0.2880 (3)	0.33726 (12)	0.87071 (14)	0.0519 (4)	
H4A	0.165 (3)	0.3529 (14)	0.8928 (16)	0.062*	
H4B	0.330 (3)	0.2842 (14)	0.8950 (17)	0.062*	
C1	0.3737 (3)	0.30049 (11)	0.66995 (14)	0.0391 (4)	
C8	−0.0739 (3)	0.42729 (11)	0.72304 (17)	0.0449 (4)	
N3	0.1269 (2)	0.37060 (11)	0.56956 (13)	0.0519 (4)	
C5	0.8555 (3)	0.18073 (13)	0.62990 (19)	0.0531 (5)	
H5	0.941 (3)	0.1645 (13)	0.5678 (17)	0.064*	
C6	0.9189 (3)	0.16063 (13)	0.74431 (19)	0.0538 (5)	
H6	1.043 (3)	0.1340 (14)	0.7618 (17)	0.065*	
C4	0.6753 (3)	0.22542 (13)	0.60482 (17)	0.0480 (5)	
H4	0.624 (3)	0.2418 (13)	0.5236 (16)	0.058*	
N6	−0.0897 (3)	0.42970 (12)	0.83758 (16)	0.0646 (5)	
C11	−0.3997 (3)	0.51251 (15)	0.7968 (3)	0.0736 (7)	
H11	−0.519 (3)	0.5398 (17)	0.8311 (19)	0.088*	
C9	−0.2150 (3)	0.46755 (14)	0.6418 (2)	0.0603 (6)	
H9	−0.190 (3)	0.4675 (14)	0.5611 (19)	0.072*	
C10	−0.3793 (3)	0.51058 (15)	0.6802 (3)	0.0711 (7)	
H10	−0.482 (3)	0.5412 (16)	0.6283 (19)	0.085*	
C12	−0.2542 (4)	0.47175 (17)	0.8721 (2)	0.0802 (8)	
H12	−0.255 (3)	0.4710 (16)	0.960 (2)	0.096*	

### Atomic displacement parameters (Å^2^)

**Table:**

	*U*^11^	*U*^22^	*U*^33^	*U*^12^	*U*^13^	*U*^23^
C2	0.0435 (10)	0.0367 (9)	0.0468 (11)	−0.0022 (8)	−0.0023 (8)	0.0011 (8)
C7	0.0573 (13)	0.0494 (12)	0.0509 (12)	0.0076 (10)	−0.0027 (10)	0.0060 (9)
N1	0.0419 (8)	0.0365 (8)	0.0364 (8)	−0.0017 (6)	0.0019 (6)	0.0009 (6)
C3	0.0403 (10)	0.0328 (9)	0.0402 (10)	−0.0060 (8)	0.0014 (7)	0.0004 (7)
N5	0.0500 (9)	0.0468 (9)	0.0420 (9)	0.0050 (7)	0.0010 (7)	0.0029 (7)
N2	0.0528 (10)	0.0531 (10)	0.0428 (9)	0.0067 (8)	−0.0035 (7)	−0.0006 (7)
N4	0.0557 (10)	0.0604 (11)	0.0400 (9)	0.0125 (9)	0.0061 (7)	0.0026 (8)
C1	0.0420 (10)	0.0379 (9)	0.0372 (10)	−0.0047 (8)	0.0025 (8)	−0.0004 (8)
C8	0.0425 (10)	0.0312 (9)	0.0610 (12)	−0.0031 (8)	0.0044 (9)	0.0017 (9)
N3	0.0539 (10)	0.0505 (9)	0.0491 (10)	0.0070 (8)	−0.0060 (7)	−0.0004 (7)
C5	0.0515 (13)	0.0498 (12)	0.0601 (13)	−0.0011 (10)	0.0172 (10)	−0.0055 (10)
C6	0.0455 (12)	0.0408 (11)	0.0749 (15)	0.0046 (9)	0.0041 (11)	0.0014 (10)
C4	0.0479 (11)	0.0531 (12)	0.0435 (11)	−0.0027 (9)	0.0076 (9)	−0.0003 (9)
N6	0.0655 (11)	0.0583 (11)	0.0734 (13)	0.0183 (9)	0.0243 (9)	0.0182 (9)
C11	0.0536 (14)	0.0501 (13)	0.121 (2)	0.0119 (11)	0.0267 (14)	0.0134 (14)
C9	0.0550 (13)	0.0480 (12)	0.0747 (15)	0.0059 (10)	−0.0096 (11)	−0.0056 (11)
C10	0.0532 (14)	0.0476 (13)	0.109 (2)	0.0097 (11)	−0.0094 (14)	−0.0053 (13)
C12	0.0793 (17)	0.0738 (16)	0.0932 (19)	0.0264 (14)	0.0378 (15)	0.0253 (15)

### Geometric parameters (Å, °)

**Table:**

C2—N3	1.319 (2)	C8—N6	1.328 (2)
C2—N1	1.361 (2)	C8—C9	1.388 (3)
C2—C8	1.468 (2)	C5—C4	1.369 (3)
C7—N5	1.342 (2)	C5—C6	1.371 (3)
C7—C6	1.372 (3)	C5—H5	0.979 (19)
C7—H7	0.988 (19)	C6—H6	0.92 (2)
N1—C1	1.358 (2)	C4—H4	0.990 (18)
N1—N4	1.410 (2)	N6—C12	1.345 (3)
C3—N5	1.337 (2)	C11—C10	1.358 (3)
C3—C4	1.386 (2)	C11—C12	1.369 (3)
C3—C1	1.470 (2)	C11—H11	1.00 (2)
N2—C1	1.321 (2)	C9—C10	1.367 (3)
N2—N3	1.367 (2)	C9—H9	0.96 (2)
N4—H4A	0.91 (2)	C10—H10	0.97 (2)
N4—H4B	0.87 (2)	C12—H12	1.01 (2)
			
N3—C2—N1	109.53 (16)	C2—N3—N2	107.73 (14)
N3—C2—C8	123.05 (16)	C4—C5—C6	119.04 (19)
N1—C2—C8	127.35 (17)	C4—C5—H5	120.9 (12)
N5—C7—C6	123.87 (19)	C6—C5—H5	120.0 (12)
N5—C7—H7	114.5 (11)	C5—C6—C7	118.6 (2)
C6—C7—H7	121.6 (11)	C5—C6—H6	119.2 (12)
C1—N1—C2	105.60 (14)	C7—C6—H6	122.1 (12)
C1—N1—N4	127.52 (15)	C5—C4—C3	118.76 (18)
C2—N1—N4	126.53 (15)	C5—C4—H4	122.0 (10)
N5—C3—C4	123.26 (17)	C3—C4—H4	119.3 (11)
N5—C3—C1	117.90 (15)	C8—N6—C12	116.51 (19)
C4—C3—C1	118.84 (16)	C10—C11—C12	118.8 (2)
C3—N5—C7	116.38 (16)	C10—C11—H11	123.6 (13)
C1—N2—N3	107.48 (14)	C12—C11—H11	117.5 (13)
N1—N4—H4A	102.4 (12)	C10—C9—C8	119.1 (2)
N1—N4—H4B	109.3 (13)	C10—C9—H9	122.1 (13)
H4A—N4—H4B	114.2 (19)	C8—C9—H9	118.7 (13)
N2—C1—N1	109.65 (15)	C11—C10—C9	119.0 (2)
N2—C1—C3	122.78 (16)	C11—C10—H10	117.6 (13)
N1—C1—C3	127.55 (15)	C9—C10—H10	123.4 (13)
N6—C8—C9	122.78 (19)	N6—C12—C11	123.8 (2)
N6—C8—C2	118.20 (16)	N6—C12—H12	111.7 (14)
C9—C8—C2	119.03 (19)	C11—C12—H12	124.5 (14)
			
N3—C2—N1—C1	0.46 (18)	N3—C2—C8—C9	−2.7 (3)
C8—C2—N1—C1	177.71 (16)	N1—C2—C8—C9	−179.56 (17)
N3—C2—N1—N4	174.11 (15)	N1—C2—N3—N2	−0.10 (19)
C8—C2—N1—N4	−8.6 (3)	C8—C2—N3—N2	−177.49 (15)
C4—C3—N5—C7	1.9 (3)	C1—N2—N3—C2	−0.32 (19)
C1—C3—N5—C7	−177.18 (15)	C4—C5—C6—C7	1.2 (3)
C6—C7—N5—C3	−0.5 (3)	N5—C7—C6—C5	−1.0 (3)
N3—N2—C1—N1	0.61 (19)	C6—C5—C4—C3	0.1 (3)
N3—N2—C1—C3	−178.15 (15)	N5—C3—C4—C5	−1.7 (3)
C2—N1—C1—N2	−0.67 (19)	C1—C3—C4—C5	177.34 (16)
N4—N1—C1—N2	−174.23 (16)	C9—C8—N6—C12	1.6 (3)
C2—N1—C1—C3	178.02 (16)	C2—C8—N6—C12	−178.42 (18)
N4—N1—C1—C3	4.5 (3)	N6—C8—C9—C10	−0.9 (3)
N5—C3—C1—N2	−176.25 (16)	C2—C8—C9—C10	179.09 (18)
C4—C3—C1—N2	4.6 (2)	C12—C11—C10—C9	0.4 (4)
N5—C3—C1—N1	5.2 (2)	C8—C9—C10—C11	−0.1 (3)
C4—C3—C1—N1	−173.88 (16)	C8—N6—C12—C11	−1.3 (4)
N3—C2—C8—N6	177.36 (17)	C10—C11—C12—N6	0.3 (4)
N1—C2—C8—N6	0.5 (3)		

### Hydrogen-bond geometry (Å, °)

**Table:**

*D*—H···*A*	*D*—H	H···*A*	*D*···*A*	*D*—H···*A*
N4—H4A···N6	0.91 (2)	2.08 (2)	2.839 (2)	141.1 (17)
N4—H4B···N5	0.87 (2)	2.39 (2)	2.863 (2)	115.0 (16)
C4—H4···N5^i^	0.990 (18)	2.509 (19)	3.457 (2)	160.1 (14)
C7—H7···N3^ii^	0.988 (19)	2.58 (2)	3.444 (2)	146.2 (14)
C6—H6···Cg1^iii^	0.92 (2)	2.75 (2)	3.504 (2)	140.4 (16)
C11—H11···Cg2^iv^	1.00 (2)	2.86 (2)	3.602 (2)	131.9 (17)

Symmetry codes: (i) *x*, −*y*+1/2, *z*−1/2; (ii) *x*+1, −*y*+1/2, *z*+1/2; (iii) −*x*+1, *y*−1/2, −*z*+3/2; (iv) −*x*, *y*+1/2, −*z*+3/2.
